# Contribution of individual COPD assessment test (CAT) items to CAT total score and effects of pulmonary rehabilitation on CAT scores

**DOI:** 10.1186/s12955-018-1034-4

**Published:** 2018-10-30

**Authors:** Sarah Houben-Wilke, Daisy J. A. Janssen, Frits M. E. Franssen, Lowie E. G. W. Vanfleteren, Emiel F. M. Wouters, Martijn A. Spruit

**Affiliations:** 1 Department of Research and Education, CIRO, Hornerheide 1, Horn, 6085 NM The Netherlands; 2 Centre of Expertise for Palliative Care, Maastricht University Medical Center, Maastricht, The Netherlands; 3 Department of Respiratory Medicine, Maastricht University Medical Center, Maastricht, The Netherlands; 4 COPD center, Sahlgrenska University Hospital and Institute of Medicine, Gothenburg University, Gothenburg, Sweden; 5 Department of Respiratory Medicine, Maastricht University Medical Center, NUTRIM School of Nutrition and Translational Research in Metabolism, Maastricht, The Netherlands

**Keywords:** Health status, COPD assessment test, GOLD classification, Pulmonary rehabilitation

## Abstract

**Background:**

The COPD Assessment Test (CAT) contains eight items (cough, phlegm, chest tightness, breathlessness, limited activities, confidence leaving home, sleeplessness and energy). The current study aimed 1) to better understand the impact of the respiratory and non-respiratory CAT item scores on the CAT total score; and 2) to determine the impact of pulmonary rehabilitation (PR) on CAT items and CAT total score.

**Methods:**

CAT total score of ≥10 or ≥ 18 points was used to classify patients as highly symptomatic, a decrease of 2 points was considered as clinically relevant improvement. ‘Cough’, ‘phlegm’, ‘chest tightness’, ‘breathlessness’ were defined as respiratory items; ≥3 points on each item was defined as highly symptomatic.

**Results:**

In total, 497 clinically stable patients (55% male, age 64.0 (57.5–71.0) years, FEV_1_ 46.0 (32.0–63.0)% predicted, CAT total score 22.0 (17.5–26.0) points) were included. 95% had CAT score ≥ 10 points and 75% ≥18 points. Respectively, 45% and 54% of subjects scored high on 3 or 4 of the respiratory CAT items. Following PR, 220 patients (57.7%) reported an improved health status as assessed by CAT total score (− 3.0 (− 7.0–1.0) points). Change in CAT item scores ranged from 0.0 (− 1.0–0.0) to − 1.0 (− 2.0–0.0) points) with best improvements in ‘energy’ (− 1.0 (− 2.0–0.0)points).

**Conclusions:**

A substantial number of patients classified as highly symptomatic did not report a high level of respiratory symptoms, indicating that non-respiratory symptoms impact on disease classification and treatment algorithm. The impact of PR on CAT item scores varied by individual item.

**Trial registration:**

Netherlands National Trial Register (NTR3416). Registered 2 May 2012.

**Electronic supplementary material:**

The online version of this article (10.1186/s12955-018-1034-4) contains supplementary material, which is available to authorized users.

## Background

Improvement in health status in patients with chronic obstructive pulmonary disease (COPD) is one of the treatment objectives as recommended by the Global initiative for chronic Obstructive Lung Disease (GOLD) committee [1]. Health status can easily be measured in patients with COPD using the COPD Assessment Test (CAT) [2]. Indeed, the CAT contains eight items, which focus on respiratory symptoms, such as cough, sputum production, chest tightness and dyspnea, but also on non-respiratory symptoms, such as lack of energy or sleep disturbance as well as additional indicators, such as limitations in doing activities at home or confidence leaving home [2]. The GOLD 2018 report recommends a CAT score of 10 points or higher to classify patients with COPD as highly symptomatic [1, 3]. A recent patient-level pooled analysis including more than 18,000 patients with COPD suggested a CAT score of 18 points or higher to classify patients as highly symptomatic [4].

A decrease in CAT score of two points is considered a clinically relevant improvement [5, 6]. Hence, the CAT has become a prominent patient reported outcome measure for patients with COPD.

To date, it remains unknown whether and to what extent the eight CAT items are related to the CAT total score. This is potentially clinically important information, as the CAT total score determines the GOLD classification of patients with COPD and, in turn, the recommended pharmacological treatment strategy [1]. However, in theory, patients with COPD can have a CAT total score of 10 points or higher not directly related to their respiratory condition, such as sleep disturbance and lack of energy, which are most probably not directly affected by the currently available respiratory drug therapies.

Statistically significant and clinically relevant improvements in health status as assessed by CAT have been reported in patients with COPD following pulmonary rehabilitation (PR) [6–8]. As PR is a comprehensive intervention with impact on all CAT items [9], improvements seem reasonable to expect. The mean change in CAT total score following PR, however, is approximately three points [6–8], suggesting that patients with COPD do not report improvements on all CAT items following PR. A detailed analysis of the impact of PR on the eight CAT items will encourage a better understanding of the true impact of PR on patients’ health status.

Therefore, this study aimed: 1) to better understand the impact of the respiratory and non-respiratory CAT item scores on the CAT total score in patients referred for PR; and 2) to determine the impact of PR on the eight CAT items and the CAT total score.

## Methods

Data were retrieved from the COPD, health status and co-morbidities (Chance) study, a longitudinal observational single-center study [10] which was approved by the local ethics committee of Maastricht University Medical Centre+, The Netherlands (MEC11–3-070). Data from the Chance study has been published previously [6, 11–19]. The change in CAT total score following PR has recently been described [6].

### Study subjects

Patients were recruited at CIRO (Horn, The Netherlands) during their pre-PR assessment between April 2012 and September 2014. Patients were eligible if they had a primary diagnosis of COPD and were clinically stable for at least 4 weeks preceding enrolment. Patients were excluded if they had a history of other lung diseases, had undergone lung surgery or had a malignancy within the last 5 years. All patients gave written informed consent. Patients eligible for PR participated in an inpatient PR program of 8 weeks or an outpatient PR program of 16 weeks (40 sessions in both settings), followed by a post PR-assessment. In brief, CIRO provides a state-of-the-art interdisciplinary PR programme [20] in line with the 2013 American Thoracic Society/European Respiratory Society Statement on PR [9]. Based on the degree of complexity, a modular treatment program is composed [21].

### Clinical characteristics

Demographic and clinical characteristics were assessed as described before [10]. Post-bronchodilator spirometry and 6-min walk distance (6MWD) were assessed according to international guidelines and standard operating procedures [1, 22].

### COPD assessment test

The CAT has been developed to provide a simple and reliable measure of disease-specific health status [2]. The CAT consists of eight items (cough, phlegm, chest tightness, breathlessness, limited activities, confidence leaving home, sleeplessness and energy) defined with contrasting adjectives. Item scores range from 0 to 5 points resulting in a CAT total score ranging from 0 to 40 points [2].A CAT total score of ≥10 points [1, 3] or ≥ 18 points [4] has been suggested to classify patients as highly symptomatic. The minimal clinically important difference of the CAT is 2 points [5, 6].

### Statistics

Categorical variables were reported as frequencies. Continuous variables were tested for normality using Shapiro-Wilk test and described as mean and standard deviation (SD) or median and interquartile range [IQR], as appropriate. Only patients with complete CAT data were included in the current analyses (*n* = 497 baseline, *n* = 381 follow-up). An independent sample t-test or Mann Whitney-U Test was used to compare patient characteristics between patients who completed PR and those who dropped out. To compare patients’ CAT scores before and after PR, a paired sample t-test or Wilcoxon signed-rank test was used. Categorical variables were compared using Chi-Square tests. Spearman correlation coefficients were calculated to explore the association between (change in) CAT item and total scores. To demonstrate the impact of respiratory symptoms on the CAT total score, the first four CAT items (cough, phlegm, chest tightness and breathlessness) were defined as respiratory items. As CAT items can be scored 0–5 points, a priori, a score ≥ 3 points on each item was defined as highly symptomatic. To estimate the effect size *r*, the formula *r = Z / √N* was used in which *z* is the *z*-score retrieved from Wilcoxon signed-rank test results and N is the number of total observations on which *Z* is based. Histograms of pre- and post-PR CAT item scores were used to demonstrate the (shift in) frequency distribution. Percentages of patients were compared using McNemar’s test. Bar diagrams were constructed using GraphPad Prism 5. Statistical analyses were performed using IBM SPSS statistics, Version 23.0. A *p*-value of ≤0.05 was interpreted as statistically significant.

## Results

Table 1 shows the baseline characteristics for patients with complete data at baseline (*n* = 497) as well as for patients with complete data at follow-up (*n* = 381) and those who dropped out during PR (*n* = 116). In general, patients had moderate to severe airflow obstruction and were highly symptomatic; 94% of the patients were classified in GOLD B or D. Patients who did not complete PR were more often current smokers, had a significantly worse diffusion capacity, a shorter 6MWD and reported more dyspnea compared to patients who completed PR (Table 1). Reasons for dropout have been described before [14].

**Table 1:** Baseline characteristics of patients with complete CAT data at baseline, complete pre- and post-PR CAT data and patients who dropped out

	Complete baseline *N* = 497	Complete pre- and post *N* = 381	Dropout *N* = 116
Age (years)^#^	64.0 (57.5–71.0)	64.0 (58.0–71.0)	64.0 (54.3–72.0)
Male gender, n (%)	273 (54.9)	204 (53.5)	69 (59.5)
BMI (kg/m^2^)^#^	25.6 (21.7–29.7)	25.7 (21.7–29.0)	25.1 (21.4–29.6)
GOLD stage, n (%)			
I	37 (7.4)	28 (7.3)	9 (7.8)
II	172 (34.6)	137 (36.0)	35 (30.2)
III	186 (37.4)	136 (35.7)	50 (43.1)
IV	102 (20.5)	80 (21.0)	22 (19.0)
GOLD grade^§^, n (%)			
A	14 (2.8)	10 (2.6)	4 (3.4)
B	158 (31.8)	132 (34.6)	26 (22.4)
C	14 (2.8)	11 (2.9)	3 (2.6)
D	311 (62.6)	228 (59.8)	83 (71.6)
FEV_1_ (L)^#^	1.2 (0.8–1.7)	1.1 (0.8–1.7)	1.2 (0.8–1.6)
FEV_1_ (% pred.)^#^	46.0 (32.0–63.0)	46.7 (32.0–62.5)	44.5 (31.8–64.0)
FEV_1_/FVC, %^#^	35.0 (28.0–45.9)	35.3 (27.9–44.9)	34.5 (28.0–48.9)
DLCO (mmol/min./kPa)^#1^	3.7 (3.0–5.0)	3.7 (3.0–5.1)	3.6 (2.6–4.8)
DLCO (%)^#1^	46.0 (37.0–59.0)	47.0 (38.0–60.1)	44.9 (34.8–56.8)*
Current smokers, n (%)^2^	110 (22.1)	71 (18.6)	39 (33.9)*
Pack years^#3^	40 (30–50)	40 (30–50)	40 (30–50)
LTOT, n (%)	120 (24.1)	94 (24.7)	26 (22.4)
6MWD, m^4^	425 (123)	436 (119)	391 (133)*
mMRC, n (%)^4^			
0	10 (2.0)	10 (2.7)	0 (0.0)
1	81 (16.5)	61 (16.2)	20 (17.4)
2	187 (38.0)	157 (41.6)	30 (26.1)*
3	124 (25.2)	83 (22.0)	41 (35.7)*
4	90 (18.3)	66 (17.5)	24 (20.9)
CAT score, points^#^			
Total score	22.0 (17.5–26.0)	22.0 (17.0–26.0)	22.0 (18.0–27.0)
Cough	3.0 (2.0–3.0)	3.0 (2.0–3.0)	3.0 (2.0–3.0)
Phlegm	2.0 (1.0–3.0)	2.0 (1.0–3.0)	2.0 (1.0–3.0)
Chest tightness	1.0 (0.0–2.0)	1.0 (0.0–2.0)	1.0 (0.0–3.0)
Breathlessness	4.0 (4.0–5.0)	4.0 (4.0–5.0)	5.0 (3.3–5.0)
Limited activity	3.0 (2.0–4.0)	3.0 (2.0–4.0)	3.0 (2.0–5.0)
Confidence leaving home	2.0 (1.0–3.0)	2.0 (1.0–3.0)	2.0 (1.0–3.0)
Sleeplessness	3.0 (1.0–4.0)	3.0 (1.0–4.0)	3.0 (1.0–4.0)
Energy	4.0 (3.0–4.0)	4.0 (3.0–4.0)	3.0 (3.0–4.0)
CAT total score ≥ 10 points, n (%)	469 (94.4)	360 (94.5)	109 (94.0)
CAT total score ≥ 18 points, n (%)	373 (75.1)	285 (74.8)	88 (75.9)

Data are presented as mean ± SD, median (interquartile range) or N (%). ^#^Not normally distributed. *Abbreviations: BMI* Body Mass Index, *COPD* Chronic Obstructive Pulmonary Disease, *GOLD* Global initiative for chronic Obstructive Lung Disease, *FEV*_*1*_ Forced Expiratory Volume in 1 s., *FVC* Forced Vital Capacity, *DLCO* Diffusing capacity of the Lung for Carbon Monoxide, *LTOT* Long Term Oxygen Therapy, *6MWD* 6 min walk distance; mMRC, modified Medical Research Council

*compared with completers, *p* ≤ 0.05

^§^according to GOLD 2018 [1], CAT used as symptom measure

^1^*n* = 456; ^2^ *n* = 496; ^3^ *n* = 476; ^4^ *n* = 492

### CAT scores

The median CAT total score was 22.0 (17.5–26.0) points. Patients reported high scores on CAT item ‘breathlessness’ (4.0 (4.0–5.0) points) while low scores were reported on CAT item ‘chest tightness’ (1.0 (0.0–2.0) points) (Table 1). Correlations between baseline CAT item scores and CAT total scores range between 0.481 and 0.649 (all *p* ≤ 0.001) (Additional file 1: Figure S1).

**Fig. 1:**
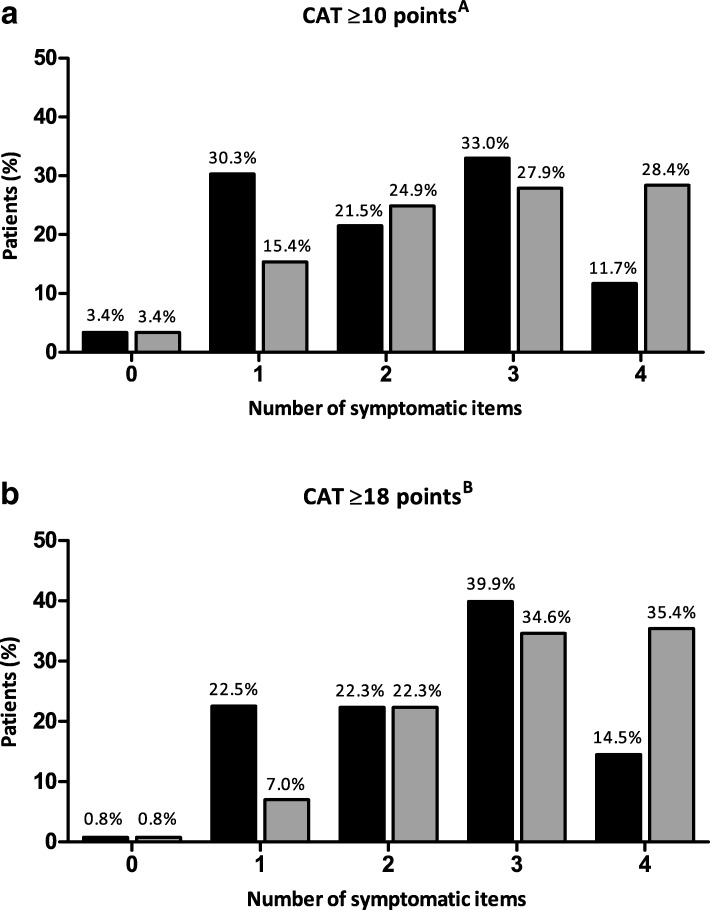
Impact of the four respiratory items (cough, phlegm, chest tightness, breathlessness; ) and four non-respiratory items (limited activity, confidence leaving home, sleeplessness, energy;) on the CAT total score a) ≥10 points (*n* = 469) and b) ≥18 points (*n* = 373). X-as demonstrates the number of symptomatic (defined as ≥3 points) CAT items. ^A^ GOLD 2018 report [1]; ^B^ Smid et al. JAMDA 2017 [4]

At baseline, 469 patients (94.4%) had a CAT total score of ≥10 points; and 373 patients (75.1%) had a CAT total score of ≥18 points. Figure 1 demonstrates the impact of the four respiratory items (cough, phlegm, chest tightness, breathlessness) as well as the four non-respiratory items (limited activity, confidence leaving home, sleeplessness, energy) on the CAT total score: of those patients reporting a CAT total score of ≥10 points, 44.7% reported a high level of symptoms on 3 or 4 of the respiratory items while 56.3% reported a high level of symptoms on 3 or 4 of the non-respiratory items (Fig. 1a). Of those patients reporting a CAT total score of ≥18 points, 54.4% reported a high level of symptoms on 3 or 4 of the respiratory items while 70.0% reported a high level of symptoms on 3 or 4 of the non-respiratory items (Fig. 1b). Scatterplots further demonstrate that patients with a CAT total score of ≥10 or ≥ 18 points can score low on respiratory items (Additional file 1: Figure S1).

### Impact of PR

**Fig. 2:**
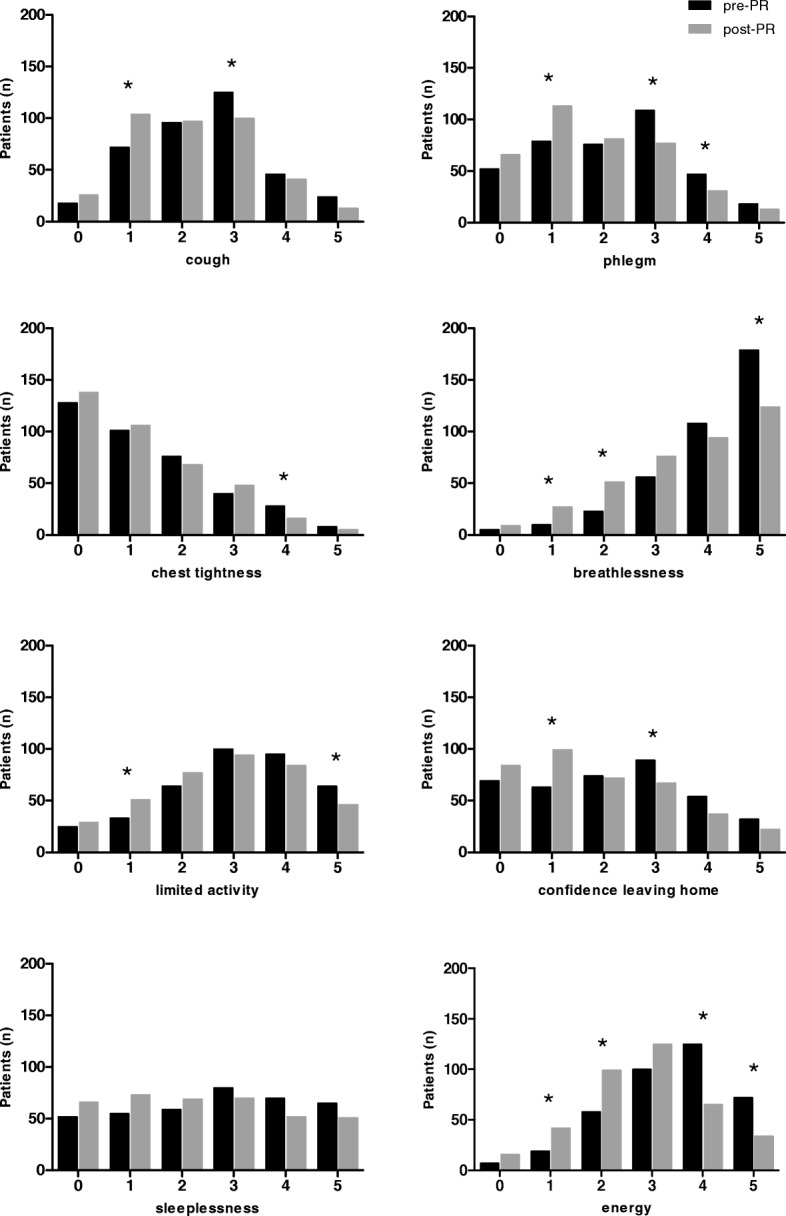
Frequency distributions of CAT item scores before and after PR (*n* = 381), * *p* ≤ 0.05

Following PR, 220 patients (57.7%) reported an improved health status (a decrease of 2 points or more) as assessed by CAT total score (change − 3.0 (− 7.0–1.0) points). CAT item scores changed ranging from − 0.0 (− 1.0–0.0) to − 1.0 (− 2.0–0.0) points) (Table 2). Changes in CAT item scores varied by individual item (Additional file 1: Table S1). Frequency distributions demonstrate an overall shift from higher (worse health status) to lower (better health status) scores following PR for all item scores (Fig. 2). Patients reported best improvements in items ‘breathlessness’ and ‘energy’ following PR (Table 2). Indeed, effect sizes for these items were the best (*r* = − 0.354 and − 0.403, respectively) (Table 2).

**Table 2:** Pre- and post-PR CAT item and total scores, mean difference and effect sizes of CAT item and total scores

	Pre-PR	Post-PR	Difference	Effect size *r*
Cough	3.0 (2.0–3.0)	2.0 (1.0–3.0)*	0.0 (− 1.0–0.0)	− 0.254
Phlegm	2.0 (1.0–3.0)	2.0 (1.0–3.0)*	0.0 (− 1.0–0.0)	−0.283
Chest tightness	1.0 (0.0–2.0)	1.0 (0.0–2.0)*	0.0 (− 1.0–0.0)	− 0.106
Breathlessness	4.0 (4.0–5.0)	4.0 (3.0–5.0)*	0.0 (− 1.0–0.0)	−0.354
Limited activities	3.0 (2.0–4.0)	3.0 (2.0–4.0)*	0.0 (− 1.0–0.0)	− 0.210
Confidence leaving home	2.0 (1.0–3.0)	2.0 (1.0–3.0)*	0.0 (− 1.0–0.0)	−0.244
Sleeplessness	3.0 (1.0–4.0)	2.0 (1.0–4.0)*	0.0 (− 1.0–1.0)	− 0.198
Energy	4.0 (3.0–4.0)	3.0 (2.0–4.0)*	− 1.0 (− 2.0–0.0)	− 0.403
Total score	22.0 (17.5–26.0)	19.0 (14.0–23.0)*	− 3.0 (− 7.0–1.0)	−0.435

*N* = 381; * compared with Pre-PR scores, *p* ≤ 0.001

Among those patients with completed follow-up data (*n* = 381), the number of patients with a CAT total score of ≥10 points decreased from 360 (94.5%) before PR to 339 (89.0%) after PR (*p* ≤ 0.001). The number of patients who reported a CAT score of ≥18 points decreased from 285 patients (74.8%) to 219 (57.5%) (p ≤ 0.001). Correlations between changes in CAT item score and changes in CAT total scores range between 0.452 and 0.634, *p* ≤ 0.001 (Additional file 1: Figure S2).

## Discussion

The current study investigated the impact of respiratory as well as non-respiratory symptoms on the CAT total score as well as the impact of PR on the eight CAT items. Although most patients were classified as highly symptomatic as categorized by a CAT total score of ≥10 points, fewer than half of the patients did not report a high level of symptoms on CAT items ‘cough’, ‘phlegm’, ‘chest tightness’ and ‘breathlessness’. Furthermore, health status as assessed by CAT total score generally improved while the impact of PR on CAT item scores varied by individual item.

### CAT in GOLD

The current study demonstrates that CAT items contribute differently to the CAT total score; items ‘breathlessness’ and ‘energy’ scored highest, while chest tightness scored lowest. Since the studied population experienced a high level of dyspnea (more than 80% have an mMRC score of ≥2 points), the substantial impact of breathlessness is not surprising. A previous study defined the CAT item ‘breathlessness’ as a predominant symptom and concluded that the predictive value of the CAT total score was overwhelmed by the breathlessness component [23]. However, the majority of patients were classified as mild to moderate COPD [23]; indeed, Jones and colleagues concluded that the item ‘breathlessness’ has greatest discriminant power for milder patients while ‘confidence leaving home’ better discriminates in more severe patients [2].

As the CAT total score determines the GOLD classification of patients with COPD (classifying patients into low (A/C) and high (B/D) symptom groups) and, in turn, the recommended respiratory pharmacological treatment strategy [1], it is important to understand the compilation of the CAT total score and the contribution of the separate items. The correlations between item and total scores were generally good. However, of those patients reporting a CAT total score of ≥10 points, only 44.7% reported a higher level of symptoms (≥3 points) on 3 or 4 of the respiratory items ‘cough’, ‘phlegm’, ‘chest tightness’ and ‘breathlessness’ items. Using the recently suggested cut-point of CAT ≥18 points [4], the percentage increases to 54.4% further supporting the idea of redefining the current cut-point of ≥10 points. Thus, a higher total score cut-off point is more related to a higher amount of respiratory symptoms which guides pharmacological COPD management according to the GOLD strategy document.

A recent study among ever-smokers with normal lung function hypothesized that the four respiratory CAT items might have a similar discriminative ability compared to CAT total score [24]. The authors used a threshold of ≥7 points for the four respiratory items and concluded that these items identified high-risk symptomatic individuals to the same extent as the threshold of the CAT total score of ≥10 points [24]. However, focusing on respiratory items only overlooks the multidimensional, systemic approach of COPD the CAT was originally intended to tackle [2]. But given the high (individual) variability in compilation of CAT total scores, using a CAT total score ≥ 10 points may be too generic to classify patients and consequently treat respiratory symptoms. A future approach might yield a respiratory and non-respiratory subdomain for the CAT score, similarly to subdomains seen in St. George Respiratory Questionnaire [25]. Accordingly, the respiratory domain might be considered to guide pharmacological COPD management, while the total score might illustrate a more generic evaluation of the patient’s health status, prompt assessment of sleep disturbances and/or guide the use of non-pharmacologic therapies.

### CAT and PR

Although all CAT item scores significantly improved following PR, the response to PR varied by individual item. This can, for instance, be explained by 1) the varying baseline scores and/or 2) limited responsiveness of respective items and/or 3) focus/content of the personalized PR program. Furthermore, these differential results demonstrate once again the importance of an individualized approach understanding the underlying mechanisms leading to an improved health status as offered during an individualized and interdisciplinary comprehensive intervention, such as PR [9]. Spruit and colleagues further underlined the importance of a multidimensional response outcome to assess the complexity of the disease and the efficacy of PR as responses to regular outcomes have been shown to be differential [20].

Remarkably, the CAT item ‘energy’ showed the largest effect size indicating the strongest effect of PR on this item. First, this might be explained by the fact that PR aims to improve cardiorespiratory fitness consequently leading to increased exercise capacity and reduced breathlessness and fatigue [9]. Second, along with improvements in exercise capacity, PR has been shown to improve sleep quality [26] which is further supported by the current study and might explain the improvement in ‘energy’. Third, an individualized PR program has been shown to improve domestic function and daily activity levels in COPD [27]. The integration of achieved physiological improvement into relevant benefits experienced by the patient may be facilitated by occupational therapists [28], by, for instance, using energy conservation techniques [29] or walking aids [30, 31]. Thus, respective CAT items are responsive to PR which is, indeed, a comprehensive intervention with an overall impact on the patient’s health [9].

## Conclusions

CAT item scores for respiratory symptoms ‘cough’, ‘phlegm’, ‘chest tightness’ and ‘breathlessness’ contribute to a limited extent to the classification of COPD patients as highly symptomatic (CAT total score ≥ 10 and/or ≥ 18 points). Thus, with regards to the GOLD classification, we need to be aware that patients might be classified and pharmacologically treated as highly respiratory symptomatic mainly based on non-respiratory systemic symptoms, thus challenging the CAT total score as the recommended symptom measure to classify patients for pharmacologic treatment. Additionally, the impact of PR on CAT item scores varied by individual item. The findings underline once again the importance of an individualized approach understanding the underlying mechanisms leading to alterations in health status in COPD.

## Additional file

Additional file 1: **Table S1.** Percentage of patients reporting an improvement in CAT items by patients reporting a decline or improvement in CAT items following PR. **Figure S1.** Correlations between baseline CAT item and total scores. **Figure S2.** Correlations between changes in CAT item scores and changes in CAT total scores. (DOCX 602 kb)

## Abbreviations

6MWD: 6-Minute Walk Distance
BMI: Body Mass Index
CAT: COPD Assessment Test
COPD: Chronic Obstructive Pulmonary Disease
DLCO: Diffusing capacity of the Lung for Carbon Monoxide
FEV_1_: Forced Expiratory Volume in 1 s
FVC: Forced Vital Capacity
GOLD: Global initiative for chronic Obstructive Lung Disease
IQR: Interquartile Range
LTOT: Long Term Oxygen Therapy
mMRC: modified Medical Research Council
PR: Pulmonary Rehabilitation
SD: Standard Deviation
