# Supplementary material for: Contribution of individual COPD assessment test (CAT) items to CAT total score and effects of pulmonary rehabilitation on CAT scores
Source: Health Qual Life Outcomes. 2018 Oct 30;16:205. doi: 10.1186/s12955-018-1034-4 (PMC6208036; doi:10.1186/s12955-018-1034-4)
Supplement: Supplementary file 1 — Table S1. Percentage of patients reporting an improvement in CAT items by patients reporting a decline or improvement in CAT items following PR. Figure S1. Correlations between baseline CAT item and total scores. Figure S2. Correlations between changes in CAT item scores and changes in CAT total scores. (DOCX 602 kb) [file 12955_2018_1034_MOESM1_ESM.docx]

**ONLINE SUPPLEMENT**

**Contribution of individual COPD Assessment Test (CAT) items to CAT total score and effects of pulmonary rehabilitation on CAT scores**

Sarah Houben-Wilke^1^, Daisy J.A. Janssen^1,2^, Frits M.E. Franssen^1,3^, Lowie E.G.W. Vanfleteren^1,3^, Emiel F.M. Wouters^1,3^, Martijn A. Spruit^1,4^

**AFFILIATIONS**

^1^ Department of Research and Education, CIRO, Horn, The Netherlands

^2^ Centre of Expertise for Palliative Care, Maastricht University Medical Center, Maastricht, The Netherlands

^3^ Department of Respiratory Diseases, Maastricht University Medical Center, Maastricht, The Netherlands

^4^ Department of Respiratory Medicine, Maastricht University Medical Centre, NUTRIM School of Nutrition and Translational Research in Metabolism, Maastricht, The Netherlands

**CORRESPONDENCE**

Dr. Sarah Houben-Wilke

Hornerheide 1

6085 NM Horn

The Netherlands

**E** sarahwilke@ciro-horn.nl | **T** +31 (0)475 587 602

|  | **% of patients with decline (≥ 1 point) and improvement (≤ -1 point)** | | | | | | | | | | | | | | | | |
| --- | --- | --- | --- | --- | --- | --- | --- | --- | --- | --- | --- | --- | --- | --- | --- | --- | --- |
| **Improved (≤ -1 point) CAT items, n** | *CAT items* | cough | | phlegm | | chest tightness | | Breathlessness | | limited activity | | confidence leaving home | | sleeplessness | | energy | |
|  | cough  (n=150) | 0% | 100% | 32% | 68% | 56% | 44% | 49% | 51% | 50% | 50% | 47% | 53% | 48% | 52% | 38% | 62% |
|  | phlegm  (n=165) | 38% | 61% | 0% | 100% | 58% | 42% | 47% | 53% | 51% | 49% | 52% | 48% | 49% | 50% | 39% | 61% |
|  | chest tightness (n=132) | 50% | 50% | 47% | 53% | 0% | 100% | 45% | 55% | 48% | 52% | 43% | 57% | 45% | 55% | 35% | 65% |
|  | breathlessness (n=171) | 56% | 44% | 49% | 51% | 57% | 43% | 0% | 100% | 43% | 57% | 43% | 57% | 52% | 49% | 34% | 66% |
|  | limited activity (n=162) | 54% | 46% | 50% | 50% | 57% | 43% | 40% | 61% | 0% | 100% | 41% | 59% | 44% | 56% | 32% | 68% |
|  | confidence leaving home (n=172) | 54% | 46% | 54% | 46% | 56% | 44% | 44% | 56% | 45% | 55% | 0% | 100% | 46% | 54% | 30% | 70% |
|  | sleeplessness  (n=163) | 52% | 48% | 49% | 52% | 55% | 45% | 49% | 51% | 44% | 56% | 42% | 57% | 0% | 100% | 38% | 62% |
|  | energy  (n=215) | 57% | 43% | 53% | 47% | 60% | 40% | 47% | 53% | 49% | 51% | 44% | 56% | 53% | 47% | 0% | 100% |

**e-table 1.** Percentage of patients reporting an improvement in CAT items (rows) by patients reporting a decline (red) or improvement (green) in CAT items (columns) following PR (n=381) (for example, 51% of the patients who improved on CAT item ‘cough’ also improved on CAT item ‘breathlessness’).

**FIGURE LEGEND**

**e-figure 1.** Correlations between baseline CAT item and total scores (n=497); black dots represent individual patients, grey boxes indicate patients classified as highly symptomatic (CAT total score ≥10 points) but with low scores (<3 points) on individual CAT items.

**e-figure 2.** Correlations between changes in CAT item scores and changes in CAT total scores (n=381); black dots represent individual patients, grey boxes indicate patients with a clinically relevant improvement (≤-2 points) in CAT total as well as CAT item scores.

**e-figure 1.**

**e-figure 2.**
